# Physiologic osteoclasts are not sufficient to induce skeletal pain in mice

**DOI:** 10.1002/ejp.1662

**Published:** 2020-10-12

**Authors:** Larissa de Clauser, Sonia Santana‐Varela, John N Wood, Shafaq Sikandar

**Affiliations:** ^1^ Molecular Nociception Group, Wolfson Institute for Biomedical Research University College London London UK; ^2^ Wolfson Centre for Age‐Related Diseases Institute of Psychiatry, Psychology and Neuroscience (IoPPN) King's College London London UK; ^3^ William Harvey Research Institute Barts and the London School of Medicine & Dentistry Mary University of London London UK

## Abstract

**Background:**

Increased bone resorption is driven by augmented osteoclast activity in pathological states of the bone, including osteoporosis, fracture and metastatic bone cancer. Pain is a frequent co‐morbidity in bone pathologies and adequate pain management is necessary for symptomatic relief. Bone cancer is associated with severe skeletal pain and dysregulated bone remodelling, while increased osteoclast activity and bone pain are also observed in osteoporosis and during fracture repair. However, the effects of altered osteoclast activity and bone resorption on nociceptive processing of bone afferents remain unclear.

**Methods:**

This study investigates whether physiologic osteoclasts and resulting changes in bone resorption can induce skeletal pain. We first assessed correlation between changes in bone microarchitecture (through µCT) and skeletal pain using standardized behavioural phenotyping assays in a mouse model of metastatic bone cancer. We then investigated whether increased activity of physiologic osteoclasts, and the associated bone resorption, is sufficient to induce skeletal pain using mouse models of localized and widespread bone resorption following administration of exogenous receptor activator of nuclear factor kappa‐B ligand (RANKL).

**Results:**

Our data demonstrates that mice with bone cancer exhibit progressive pain behaviours that correlate with increased bone resorption at the tumour site. Systemic RANKL injections enhance osteoclast activity and associated bone resorption, without producing any changes in motor function or pain behaviours at both early and late timepoints.

**Conclusion:**

These findings suggest that activation of homeostatic osteoclasts alone is not sufficient to induce skeletal pain in mice.

**Significance statement:**

The role of osteoclasts in peripheral sensitization of sensory neurones is not fully understood. This study reports on the direct link between oestrogen‐independent osteoclast activation and skeletal pain. Administration of exogenous receptor activator of nuclear factor kappa‐B ligand (RANKL) increases bone resorption, but does not produce pro‐nociceptive changes in behavioural pain thresholds. Our data demonstrates that physiologic osteoclasts are not essential for skeletal pain behaviours.

## 1 INTRODUCTION

Several pathological bone conditions, including metastatic bone cancer, osteoporosis and fracture repair, are characterized by increased bone resorption and structural changes in bone microarchitecture. Pain is frequently the biggest symptomatic burden in these bone pathologies, but the effect of altered osteoclast activity on nociceptive processing remains unclear.

Cancer‐induced bone pain (CIBP) is a unique and complex condition that occurs as result of primary tumours metastasizing to the bone and involves mechanisms that are characteristic of inflammation, neuropathy, nerve compression and ischaemia (Falk & Dickenson, 2014; Honore & Mantyh, 2000). In animal models of CIBP osteoclast activity is increased (Currie et al., 2013). Pharmacological agents that target osteoclasts include bisphosphonates and denosumab, the latter an inhibitor of receptor activator of nuclear factor kappa‐B ligand (RANKL), which is involved in osteoclast differentiation (Kong et al., 1999; Matsuzaki et al., 1998). Both bisphosphonates and denosumab are clinically licensed drugs that reduce bone loss, and in some cancer patients also relieve pain (Body et al., 2004; Lipton & Balakumaran, 2012).

In humans, osteoporotic pain is often only present after vertebral fractures and resolves after healing (Kim & Vaccaro, 2006), but the relationship between bone integrity and pain outcomes is inconsistent (Li et al., 2010). While in osteoarthritic patients, pain correlates with reduced tibial bone mineral density (BMD; Burnett et al., 2017), low back pain is associated with increased lumbar BMD (Lee et al., 2013). Models of osteoporosis involving ovariectomized rodents show mechanical hyperalgesia (Li et al., 2014; Sanoja & Cervero, 2005) and reduced BMD, which can both be reversed by targeting osteoclast activity with bisphosphonate therapy (Abe et al., 2015).

The initial, sharp pain produced by fractures in humans (Santy & Mackintosh, 2001) is generally reduced by repositioning and immobilization of the fractured bone (Camuso, 2006; Crandall et al., 2002). Similarly, in rodent fracture models a reduction in weight bearing on the affected limb is observed (Jimenez‐Andrade et al., 2009). During fracture healing, osteoclasts show low‐grade activity already during the early inflammatory phase of bone remodelling, which persists to increase for the duration of the healing process (Schell et al., 2006). Prolonged administration of bisphosphonates in rats impairs fracture healing (Savaridas et al., 2013), whereas a single bolus does not delay fracture repair (McDonald et al., 2008). These observations suggest that osteoclast activity is important throughout the healing process. The temporal role of osteoclast‐mediated bone resorption during fracture repair, osteoporosis and bone cancer pain suggests that osteoclasts may play a pro‐nociceptive role or mediate the resolution of pain in bone pathologies.

The aim of this study was to determine the contribution of osteoclasts to bone pain. We hypothesized that physiologic osteoclast activity alone is sufficient to induce skeletal pain. We first determined the extent of bone resorption and pain behaviours in a syngeneic model of CIBP. We then administered exogenous RANKL, locally within the femur and systemically, to induce bone resorption through physiologic osteoclast activation and assessed the effects on pain behaviour.

## 2 MATERIALS AND METHODS

### 2.1 Cell culture

LL/2 Lewis Lung carcinoma cells (ATCC) were cultured in DMEM supplemented with 10% of FBS and 1% of Penicillin/Streptomycin for at least 2 weeks prior to surgery. Cells were split at 70%–80% confluence 1–2 days prior to surgery (cell culture reagents supplied by Thermo Fisher). On the day of surgery cells were harvested with 0.05% of Trypsin‐EDTA, resuspended in DMEM at a final concentration of 2 × 10^7^ cells/ml and kept on ice till use. Cells were counted before and after intrafemoral injection to confirm viability.

### 2.2 Mice

A total of 88 C57Bl/6J mice (80 male and 8 female, 10–14 weeks old) purchased from Charles River and Envigo were used in this study. Female mice were used only for the systemic RANKL injection model to assess changes in BMD after 24 hr. Mice were housed in groups of 2–5 mice with a 12 hr light/dark cycle and allowed free access to water and standard diet. All mice were acclimatized for 2 weeks before the start of the experiment and randomized in experimental groups across cages. All experiments were performed with approval of personal and project licenses from the United Kingdom Home Office according to guidelines set by the Animals (Scientific Procedures) Act 1986 Amendment Regulations 2012, as well as guidelines of the Committee for Research and Ethical Issues of IASP.

### 2.3 Surgery

We used an established model of CIBP involving administration of cancer cells into the distal femoral marrow (Bangash et al., 2018; Huang et al., 2008; Isono et al., 2011; Minett et al., 2014; Yang et al., 2018). Briefly, mice were anaesthetized either with a mixture ketamine (125 mg/kg)/xylazine (13 mg/kg) (late stage cancer) or isoflurane (all other experiments) and sterile lacri‐lube applied to their eyes. An incision was made in the skin above the patella. The patella and the lateral retinaculum tendons were loosened to move the patella to the side and expose the distal femoral epiphysis (Falk et al., 2013). A 30G needle was used to make a hole through the growth plate till the medullary cavity was reached. The needle was removed and a 30G syringe was used to introduce 10 μl of either 2 × 10^5^ carcinoma cells in DMEM medium, 10 μg RANKL (human recombinant RANKL, Oriental Yeast Co., 47187000) in 1 mM of EDTA in PBS or vehicle. The hole was closed with bone wax (Johnson & Johnson) and the wound irrigated with sterile saline. The patella was moved back in place. The skin was sutured with 6–0 absorbable vicryl rapid (Ethicon) and lidocaine spray (Intubeaze, 20 mg/ml, Dechra) was applied to the superficial incision site.

### 2.4 RANKL administration

To reproduce the previously described widespread bone loss model (Tomimori et al., 2009), mice were administered 1 or 2 mg/kg RANKL (or 1 mM EDTA in PBS for the vehicle group) injected intraperitoneally daily for 2 days. As RANKL promotes the migration of osteoclast precursors towards bone (Kotani et al., 2013), we also used RANKL in a priming paradigm where mice were primed with an i.p. injection of 2 mg/kg RANKL, followed by a local intrafemoral injection of 10 μl RANKL after 24 hr. Mice in the vehicle group were injected with 1 mM of EDTA in PBS.

### 2.5 Behavioural tests

For behavioural experiments, mice were acclimatized to the equipment for at least 2 days prior to testing. The experimenter was blind to the groups. For experiments involving intrafemoral injections, any mice with a limb score (LS) of less than 3 at 4 days after surgery were excluded to ensure pain outcomes were not due to the surgical procedure. Based on the criterion, we excluded the following mice: two animals for late stage cancer, one animal for intrafemoral RANKL injection, one animal for the priming i.p. followed by intrafemoral RANKL injection. Additionally, one animal from intermediate stage cancer was excluded as it failed to develop pain behaviour in the 20 day time period. This animal did not show a reduction in BMD.

#### 2.5.1 Limb use score

Mice were allowed to freely move around in a glass box (30 × 45 cm) for 5–10 min of acclimatization. Then, each mouse was observed for a period of 4 min and the use of the affected limb was scored as previously described (Falk et al., 2013): 4 = Normal use of the limb; 3 = slight limping, characterized by preferential use of the unaffected limb when rearing; 2 = clear limping; 1 = clear limping and partial lack of use of the limb; 0 = lack of use of the affected limb during most of the observation time. LS = 2 was used as endpoint for intermediate and LS = 0 for late stage cancer.

#### 2.5.2 Weight Bearing

Changes in weight bearing were assessed using an Incapacitance Metre (Linton Instrumentation) consisting of two scales. The mouse was allowed to place its head and upper body into a plastic tube to reduce stress and the hind limbs were positioned each on one of the scales. The load of each limb on the scale was measured for 5 s in which the mouse was still. Measurements were taken in triplicate, changing the position of the hind legs after each trial. The average weight‐bearing ratio was calculated as the weight placed on the affected limb (left paw) divided by the total weight on both hind limbs:

$$\frac{\sum_{i=1}^{3}\left(\frac{{\text{weight}}_{\text{left}}}{{\text{weight}}_{\;\text{left}\;}+{\text{weight}}_{\text{right}}}\right)}{3}\times 100$$

#### 2.5.3 Rotarod

The accelerating rotarod test is a behavioural paradigm to assess motor coordination (Jones & Roberts, 1968). Mice were placed on top of an accelerating rod (Life Science Series 8) and the rotation speed was set at 4 rpm. This speed was maintained for 30 s to allow mice to acclimatize. Time spent on the rotarod was recorded from the end of this period, at which point the speed was increased from 4 to 40 rpm in a 3 min ramp with a cut‐off point of 5 min from the start of acceleration. The time was recorded till the animal fell from the rotarod. The test was repeated three times and the times averaged for each animal.

#### 2.5.4 von Frey

Mechanical hypersensitivity was measured using the up‐down method to obtain the 50% withdrawal threshold (Chaplan et al., 1994). In brief, mice were placed in darkened enclosures with wire mesh floor and left to habituate for at least 1 hr till movement was reduced to a minimum. Filaments were applied perpendicular to the plantar surface for 3 s. Interval between stimuli was at least 30 s. Starting from a 0.4 g filament, the response was recorded as negative for no reaction or as positive for paw withdrawal. A positive response resulted in a decrease in filament strength for the next stimulation, a negative response in increased strength. To determine the optimal threshold six responses in proximity of the 50% threshold are required. Thus, starting from the point at which the response to a filament changed from positive to negative or negative to positive, five further responses were recorded. When continuous positive responses were observed to the minimum stimulus of 0.008 g this set cut‐off was used as the 50% withdrawal threshold. In the other cases, the pattern of responses obtained was used to calculate the 50% threshold = (10[*χ* + κ*δ*])/10,000), where χ is the log of the final von Frey filament used, κ the tabular value for the pattern of responses and δ the mean difference between filaments (in log units).

#### 2.5.5 Hotplate

The mouse was placed into the hotplate apparatus (Ugo Basile), which was held at a temperature of 50°C. The test ended when the animal showed a withdrawal response or licked one of the hind paws. Cut‐off time was set to 45 s.

#### 2.5.6 Cold‐plantar test

Cold‐plantar test was used as previously reported (Brenner et al., 2012). Paw‐withdrawal latency to the application of a pellet of compressed dry ice to the affected paw through a glass surface (6 mm thick) was recorded. Time for withdrawal was measured with a cut‐off of 45 s. Testing was repeated for three times, with a waiting period of 15 min between stimulations.

### 2.6 Tissue processing

At the end of the experiment, mice were euthanized using carbon dioxide. For the CIBP model, animals were sacrificed paired with a sham when a LS of either 2 (intermediate stage) or 0 (late stage) was reached. Blood was collected through cardiac puncture, kept at RT for 30 min to 1 hr, and then, centrifuged for 10 min at 2000 g. Supernatant serum was collected and kept in −80°C till further use. Femurs were collected and fixed in 4% of PFA for 24 hr. They were then transferred to 70% of EtOH until micro‐CT scanning.

#### 2.6.1 Quantitative analysis of TRAP in serum

TRAP solution buffer was obtained by adding freshly made 50 mM of L‐Ascorbic acid, 20 mM of disodium tartrate and 80 mM of 4‐nitrophenylphosphate to TRAP reaction buffer (1 M Acetate, 0.5% Triton X‐100, 1 M NaCl, 10 mM EDTA, pH = 5.5) and ultrapure water at a ratio of 1:1:1:2:3. Serum samples were diluted 1:10 and 20 μl of each sample were loaded in triplicate into a 96 flat bottom well plate (Nunc‐Immuno). 80 μl of freshly made TRAP solution buffer was added to each well. The microplate was incubated at 37°C for 1 hr in the dark. The reaction was stopped by adding 100 μl of 0.3 M NaOH to each well. Absorbance was measured at 405 nm using a microplate reader as previously described (Karsdal et al., 2005).

#### 2.6.2 Micro‐computed tomography (µCT)

Images were acquired with SkyScan software at 6.41 µm/pixel with 0.6° rotation steps and 2 frame averaging. Image data were reconstructed with NRecon software. CT‐analyser was used to select a 1 mm volume of interest (VOI) region, starting at 0.6 mm from the growth plate of the distal femur. Separate VOIs where created for cortical (CIBP) or trabecular (all models) bone surface through manual selection. BMD was quantified by plotting attenuation coefficients against a standard curve determined with two phantoms with known density (250 and 750 mg/cm^3^). BoneJ2, a collection of skeletal biology plug‐ins for ImageJ, was used for microarchitecture analysis (Doube et al., 2010). The 1 mm VOI was binarized and standardized 3D geometric parameters were determined, including total volume (TV), total bone volume (BV), bone surface (BS), trabecular thickness (Tb.Th.) and trabecular spacing (Tb.Sp.). To determine trabecular number (Tb.N.) and connectivity density (~number of trabeculae per unit volume), the images were first purified using a chunk size of four slices and a mapped labelling algorithm, to obtain a single connected structure throughout the image stack.

### 2.7 Statistics

Using G*Power (Faul et al., 2007), we calculated sample size using two‐tailed *t*‐test, on alpha level = 0.05, power = 0.80 and effect size = 2.8406 (based on BMD from late stage cancer). We found that the minimum needed sample size was four mice per group. Statistical analysis was performed with GraphPad Prism 8. Kruskal–Wallis test was used to determine if LS at the endpoint significantly differed between groups. Unpaired Welch's *t*‐test (two‐tailed) was used to compare difference between two distributions. Comparison of multiple timepoints between groups was performed by two‐way ANOVA with Bonferroni post hoc test. For data with missing timepoints mixed effects model REML with Bonferroni post hoc test was used instead. Data are presented as mean ± standard error of the mean (SEM) and significance as: *p < .05; **p < .01; ***p < .001, ****p < .0001.

## 3 RESULTS

### 3.1 CIBP is associated with a reduction in limb use and weight bearing

**Figure 1:**
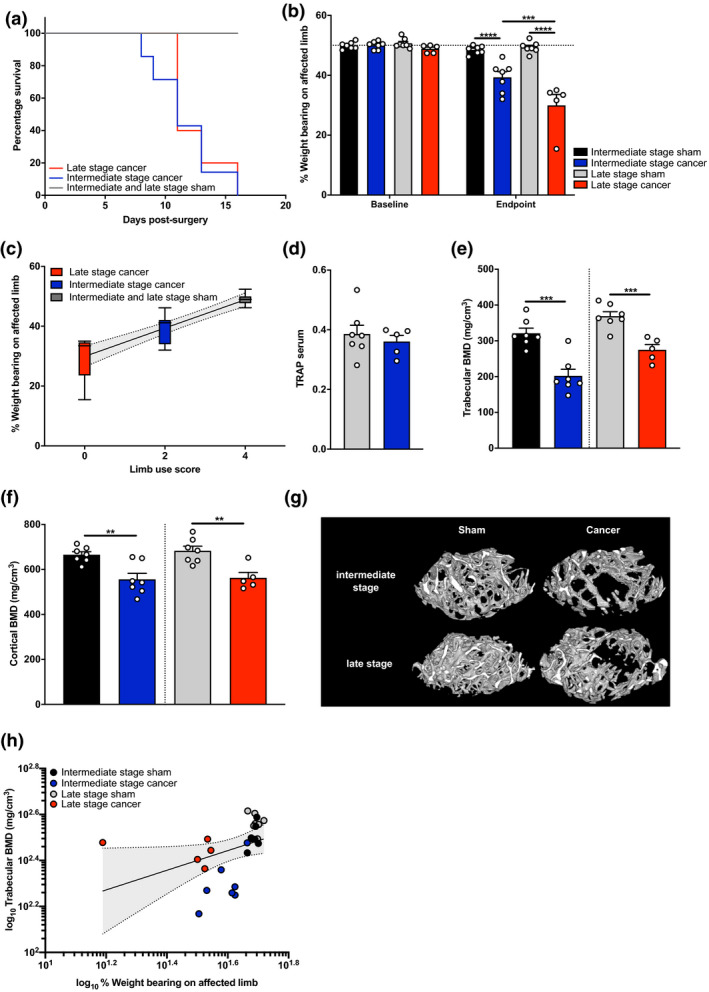
Pain behaviour and structural outcomes in a mouse model of cancer‐induced bone pain. (a) Survival curve after surgery for sham (dark grey line, *n* = 14), intermediate (blue line, *n* = 7) and late stage cancer (red line, *n* = 5) mice with endpoint defined as limping (LS = 2) and non‐use of the affected limb (LS = 0), respectively. (b) Weight bearing on the affected limb at baseline and endpoint in sham mice (black bar for intermediate stage, *n* = 7; light grey for late stage, *n* = 7), intermediate stage cancer mice (blue, *n* = 7) and late stage cancer mice (red, *n* = 5). (c) Correlation between limb use score and weight bearing, showing the median (straight line), the 25–75th percentile (boxes), the 5–95th percentile (whiskers) and interpolated linear curve with 95% confidence interval (dotted line, grey area). (d) Systemic levels of TRAP in serum of sham and late stage cancer mice. (e) Trabecular BMD and (f) cortical BMD in intermediate and late stage cancer mice. (g) Representative μCT images from sham, intermediate stage cancer and later stage cancer femurs. (h) Correlation between log transformed trabecular BMD and weight bearing on the affected limb, showing single values with interpolated linear curve (straight line) and 95% confidence interval (dotted lines). Data are shown as mean ± SEM. **p ≤ .01, ***p ≤ .001, ****p ≤ .0001 for differences between groups

We used a previously described syngeneic model of metastatic bone cancer to investigate the correlation between bone degradation and pain behaviour (Bangash et al., 2018; Huang et al., 2008; Isono et al., 2011; Minett et al., 2014; Yang et al., 2018). We used the LS of the affected hindpaw to quantify the pain phenotype associated with metastatic bone cancer. In two independent studies, we defined the endpoint either as ‘intermediate stage’ when the mice showed clear signs of limping (LS = 2) or ‘late stage’ when the use of the affected limb was completely avoided (LS = 0). At the endpoint LS significantly differed between groups (Kruskal–Wallis test: ****p < .0001). Corresponding survival curves show that mice reached a limb use score of 2 between days 8 and 15, whereas mice reached a LS of 0 at a later timepoint, between days 12 and 15 (Figure 1a). All cancer mice showed a significantly reduced survival compared to sham mice (Log‐rank (Mantel‐Cox) test: ***p = .0001). Additionally, weight bearing on the affected limb was assessed. Both intermediate and late stage cancer mice showed a significant reduction in weight bearing on the affected limb compared to sham mice when they reached their respective endpoints of the study (Figure 1b, two‐way ANOVA with Bonferroni post hoc: ****p < .0001). This effect was more pronounced in the late stage cancer mice (baseline versus endpoint; two‐way ANOVA with Bonferroni post hoc: ***p = .0001) and is highlighted by a significant linear correlation between limb use score and percentage weight bearing on the affected limb (Figure 1c, Pearson correlation: ****p < .0001, interpolated linear curve: *y* = 4.745*x* + 29.910, *r* ^2^ = .7595).

#### 3.1.1 Intermediate and late stage cancer are associated with bone resorption

We measured serum TRAP as a marker for increased osteoclast activity, but found no differences between late stage cancer and sham mice (Figure 1d). However, trabecular BMD was reduced by ~37% in intermediate cancer stage mice (202 ± 18.64 vs. 321.1 ± 14.39 mg/cm^3^; Welch's *t*‐test: ***p = .0003) and by ~25% in late stage cancer mice (202 ± 18.64 vs. 321.1 ± 14.39 mg/cm^3^; Welch's *t*‐test: ***p = .0010) compared to respective sham mice (Figure 1e). Cortical BMD was reduced by ~16% in intermediate stage cancer mice (555.8 ± 26.96 vs. 665.4 ± 13.17 mg/cm^3^; Welch's *t*‐test: **p = .0056) and by ~17% in late stage cancer mice (562.7 ± 28.83 vs. 682.9 ± 20.61 mg/cm^3^; Welch's *t*‐test: **p = .0042), compared to respective sham mice (Figure 1f). Representative µCT images and 3D reconstruction of the analysed 1 mm volume are shown in Figure 1g. We found a significant positive correlation between BMD and skeletal pain, the latter measured as percentage weight bearing on the affected limb (Figure 1h, Pearson correlation: *p = .0468, interpolated linear curve: *y* = 0.4278*x* + 1.759, *r* ^2^ = .1547). Sustained reductions in trabecular and cortical BMD indicate ongoing osteoclast activity that augments bone resorption throughout intermediate to late stage cancer in the mouse model.

### 3.2 A localized injection of RANKL is not sufficient to produce bone resorption and pain behaviour

**Figure 2:**
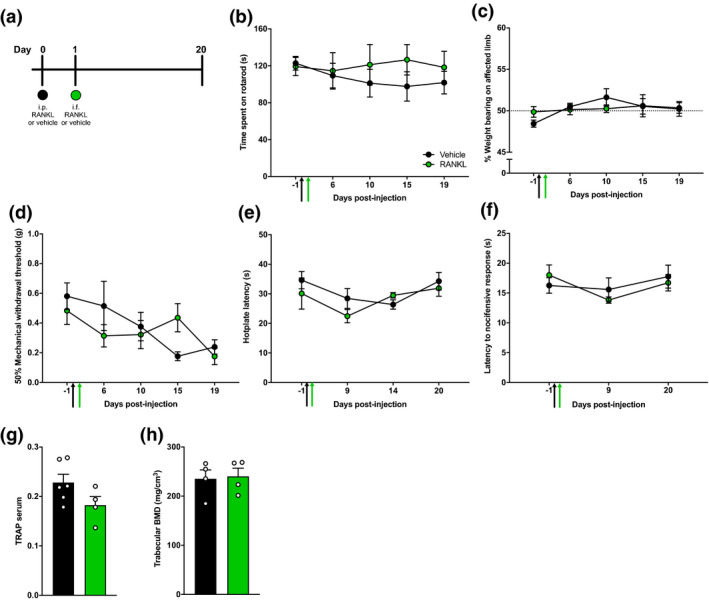
Effects of a combination of systemic and localized administration of RANKL on bone resorption and pain behaviour. (a) Schematic overview of the study design. Following a priming i.p. injection (black circle) mice received an intrafemoral injection (green circle) of RANKL or vehicle and their behaviour was closely followed for 20 days. (b) RANKL‐ (green, *n* = 4) and vehicle‐injected mice (black, *n* = 6) were assessed for motor function measured through latency to fall on a rotarod. (c) Ongoing pain was measured through weight bearing on the injected limb. (d) Mechanical sensitivity was measured by withdrawal thresholds to von Frey filaments. Thermal sensitivity was measured as nociceptive latencies to (e) noxious heat and (f) noxious cold. (g) Serum levels for the osteoclast biomarker TRAP. (i) Trabecular BMD in the ipsilateral femur. Data are shown as mean ± SEM

To investigate the role of local physiologic osteoclasts in skeletal pain, we first activated locally resident osteoclasts in the mouse femur using 10 μl of RANKL (1 mg/ml). A single intrafemoral administration of RANKL did not produce significant changes in pain behaviour or BMD (Fig. S1). Therefore, we resorted to priming mice with a systemic injection of 2 mg/kg i.p. RANKL (or vehicle) in order to promote the migration of osteoclast precursors towards bone (Kotani et al., 2013), followed by a local intrafemoral injection of 10 μl RANKL (or vehicle) on the following day (Figure 2a). Both vehicle‐ and RANKL‐injected mice showed normal motor function, assessed as time spent on rotarod (Figure 2b, two‐way ANOVA with Bonferroni post hoc, *p* = .9608). Mice did not show any changes in weight bearing on the affected limb (Figure 2c, two‐way ANOVA with Bonferroni post hoc, *p* = .4935), and did not show mechanical hypersensitivity (Figure 2d, two‐way ANOVA with Bonferroni post hoc, *p* = .6040). Similar responses to noxious heat stimulation (Figure 2e, two‐way ANOVA with Bonferroni post hoc, *p* = .3571) and noxious cold stimulation (Figure 2f, two‐way ANOVA with Bonferroni post hoc, *p* = .8013) were observed between RANKL‐ and vehicle‐injected mice. Serum levels of the bone resorption biomarker TRAP (Figure 2g, Welch's *t*‐test: *p* = .0973) and trabecular BMD (Figure 2h, Welch's *t*‐test: *p* = .8499) did not differ between RANKL‐ and vehicle‐injected mice. The results together indicate that the combination of systemic and localized RANKL administration does not produce structural changes in bone integrity or pain behaviour.

### 3.3 A model of widespread bone resorption produces sex‐specific and temporal changes in BMD

**Figure 3:**
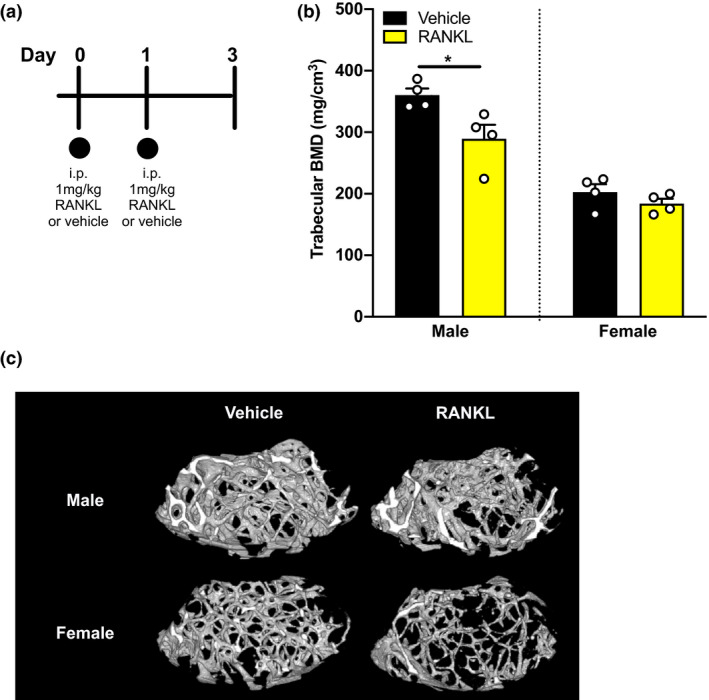
Effects of consecutive systemic administration of RANKL on bone resorption. (a) Schematic representation of the study design. Mice received two consecutive systemic injections of 1 mg/kg RANKL and bones were collected 2 days after the last injection. (b) Trabecular BMD in male and female mice injected with RANKL (yellow, *n* = 4 for female and *n* = 4 for male) or vehicle (black, *n* = 4 for female and *n* = 4 for male). (c) Representative μCT images from male and female vehicle‐ and RANKL‐injected femurs. Data are shown as mean ± SEM. *p ≤ .05 for differences between groups

**Figure 4:**
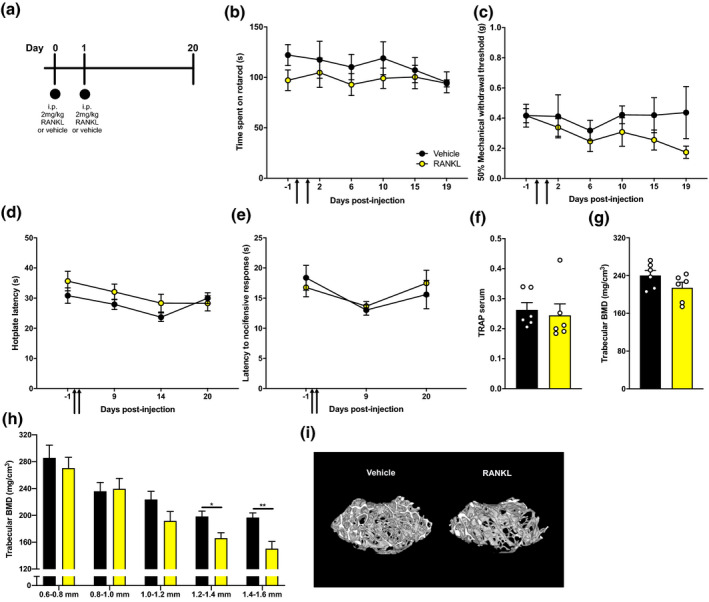
Effects of consecutive, high‐dose systemic administration of RANKL on pain behaviour and bone resorption. (a) Schematic representation of the study design. Mice received two consecutive systemic injections (black arrows) of 2 mg/kg RANKL (yellow, *n* = 6) or vehicle (black, *n* = 6). Behaviour was closely followed for 20 days, when bones were collected. (b) Mice were assessed for motor function, (c) mechanical sensitivity to von Frey filaments, (d) heat sensitivity and (e) cold sensitivity. (f) Serum levels for biomarker TRAP at 20 days. (g) Trabecular BMD of a 1 mm volumetric region starting at 0.6 mm from the growth plate. (h) Trabecular BMD for 0.2 mm thick volumetric sections at increasing distance from the growth plate. (i) Representative μCT images from vehicle‐ and RANKL‐injected femurs. Data are shown as mean ± SEM. *p ≤ .05, **p ≤ .01 for differences between groups

To determine whether the absence of bone resorption in Figure 2 was related to a low dose of RANKL used in the intrafemoral injections, we used a published murine model of widespread induction of bone resorption (Tomimori et al., 2009) that involves consecutive i.p. injections of 1 mg/kg of RANKL (Figure 3a). Forty‐eight hour after RANKL injections, we found a significant reduction in trabecular BMD in male (Welch's *t*‐test *p = .0443), but not female mice (Figure 3b; Welch's *t*‐test, *p* = .2675). As a bone resorptive phenotype was observed at 48 hr, a higher dose of 2 mg/kg RANKL was administered on two consecutive days in male mice via i.p. injection and pain behaviour was closely followed over a period of 20 days (Figure 4a). Motor behaviour was not impaired in RANKL‐injected mice compared to vehicle, as assessed through time spent on rotarod (Figure 4b, two‐way ANOVA with Bonferroni post hoc, *p* = .3365). Mechanical sensory function was not significantly different between RANKL‐ and vehicle‐injected mice (Figure 4c, mixed effects model REML with Bonferroni post hoc, *p* = .2277). Thermal pain thresholds on the hotplate (Figure 4d, two‐way ANOVA with Bonferroni post hoc, *p* = .2706) and cold‐plantar test (Figure 4e, two‐way ANOVA with Bonferroni post hoc, *p* = .8533) were similar between groups. Moreover, 20 days after two consecutive i.p. injections of RANKL, systemic levels of the biomarker TRAP in serum did not differ between RANKL‐ and vehicle‐injected mice (Figure 4f). In contrast to the observed loss of trabecular BMD at 48 hr using two RANKL i.p. administrations at a smaller dose (Figure 3), no discernible differences in overall trabecular BMD could be observed after 20 days using a higher dosing regime (Figure 4g, Welch's *t*‐test: *p* = .1328). However, 3D reconstruction of μCT data suggests that a more localized reduction in BMD was present in most mice (Figure 4h). Further stratification of the data, by dividing the analysed 1 mm volumetric region in smaller segments of 0.2 mm, revealed a significant reduction in BMD in the two segments which were further away from the growth plate (Figure 4i, Welch's *t*‐test: *p = .0183 for 1.2–1.4 mm and **p = .0057 for 1.4–1.6 mm, *p* > .1000 for the other segments).

### 3.4 Changes in bone microarchitecture is correlated with pain behaviour in a murine model of bone cancer, but not following induction of localized or widespread bone resorption

We quantified and compared changes of trabecular bone microarchitecture in cancer mice, RANKL‐injected mice and their sham/vehicle counterparts (Table 1). In mice with intermediate stage cancer, several parameters including percentage bone volume fraction (%BV/TV) (Welch's *t*‐test, ***p = .0006), bone surface fraction (BS/TV) (Welch's *t*‐test, ***p = .0003), trabecular spacing (Tb.Sp.) (Welch's *t*‐test, *p = .0416), trabecular number (Tb.N) (Welch's *t*‐test, *p = .0189) and connectivity density (Conn.D.) (Welch's *t*‐test, *p = .0139) were substantially reduced compared to sham mice. On the contrary, in late stage cancer mice, only a trend in changes of these parameters could be found. Twenty days after two consecutive systemic injections of 2 mg/kg RANKL changes in bone microarchitecture were still visible, with RANKL‐injected mice showing significantly reduced BS/TV (Welch's *t*‐test, ***p = .0006), Tb.N. (Welch's *t*‐test, ***p = .0003), Conn.D. (Welch's *t*‐test, **p = .0013), whereas trabecular thickness (Tb.Th.) was substantially increased (Welch's *t*‐test, *p = .0375) compared to vehicle‐injected mice. While not significant, similar changes were present 48 hr after two consecutive injections of 1 mg/kg RANKL, with male mice showing a substantial decrease in BV/TV (Welch's *t*‐test, **p = .0013). We also found a substantial reduction in % BV/TV (Welch's *t*‐test, **p = .0227), BS/TV (Welch's *t*‐test, **p = .0220), Tb.N. (Welch's *t*‐test, **p = .0050) and Conn.D. (Welch's *t*‐test, **p = .0069) in female mice.

**Table 1:** Parameters of bone microarchitecture in C57BL/6J receiving different agents that promote osteoclast activity

Parameter	Intermediate stage		Late stage		20 days (male)		48 hr (male)		48 hr (female)	
	Sham	Cancer	Sham	Cancer	Vehicle	RANKL	Vehicle	RANKL	Vehicle	RANKL
Cross‐section	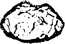	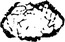	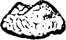	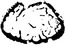	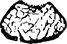	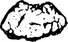	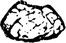	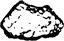	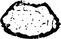	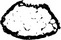
1 mm VOI	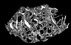	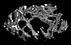	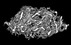	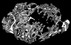	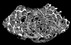	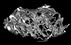	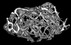	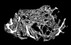	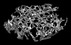	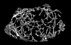
BMD	316.904 ± 11.986	205.004 ± 15.760***	368.758 ± 11.612	274.924 ± 13.148***	240.268 ± 9.890	214.152 ± 10.686	360.373 ± 9.322	289.418 ± 19.704*	202.738 ± 11.091	184.015 ± 6.735
%BV/TV	9.369 ± 0.644	4.656 ± 0.694***	10.007 ± 0.561	8.010 ± 1.021	10.061 ± 0.509	8.620 ± 0.484	10.484 ± 0.639	7.395 ± 0.768*	4.549 ± 0.270	3.359 ± 0.145*
BS/TV (mm^−1^)	6.267 ± 0.375	3.258 ± 0.413***	6.568 ± 0.397	5.593 ± 0.536	6.787 ± 0.230	5.078 ± 0.222***	6.331 ± 0.433	4.918 ± 0.311	3.759 ± 0.191	2.898 ± 0.086*
Tb.Th. (mm)	0.052 ± 0.001	0.049 ± 0.001	0.051 ± 0.001	0.048 ± 0.002	0.051 ± 0.001	0.062 ± 0.004*	0.058 ± 0.001	0.053 ± 0.003	0.042 ± 0.001	0.040 ± 0.001
Tb.Sp. (mm)	0.612 ± 0.017	0.715 ± 0.036**	0.550 ± 0.014	0.550 ± 0.019	0.564 ± 0.011	0.591 ± 0.012	0.553 ± 0.015	0.582 ± 0.006	0.544 ± 0.017	0.542 ± 0.022
Tb.*N*. (mm^−1^)	909.643 ± 205.335	207.071 ± 42.560*	634.429 ± 69.279	480.300 ± 130.107	589.500 ± 14.431	336.083 ± 32.111***	469.125 ± 46.396	399.750 ± 41.469	267.625 ± 19.513	140.875 ± 11.636**
Conn.D. (mm^−3^)	217.472 ± 44.269	54.831 ± 10.766*	171.904 ± 20.176	130.264 ± 34.817	151.946 ± 6.373	91.194 ± 9.163**	128.926 ± 11.003	111.227 ± 8.762	87.9670 ± 6.740	47.674 ± 4.333**

Note

Data are shown as mean ± standard error of the mean. For each treatment, data were compared with the respective control group and differences in distribution were determined by unpaired Welch's *t*‐test (***p ≤ .001, **p ≤ .01, *p ≤ .05). Representative pictures were chosen based on the average measured BMD and show binarized cross‐section at 1 mm from the growth plate (upper row, scalebar 500 µm) and 3D reconstruction of the 1 mm VOI (0.6–1.6 mm from growth plate) used to determine parameters for bone microarchitecture: % BV/TV (%bone volume fraction), BS/TV (bone surface fraction), Tb.Th. (trabecular thickness), Tb.Sp. (trabecular spacing), Tb.N. (number of trabeculae), Conn.D. (Connectivity density or number of trabeculae per volume).

## 4 DISCUSSION

### 4.1 Bone cancer is associated with a dysregulation in normal bone remodelling and correlates with pain behaviour

We used a syngeneic model of CIBP to determine outcomes on bone resorption and the development of chronic pain. Previous studies of CIBP have used mice at 6–8 weeks (Falk et al., 2013; Guedon et al., 2016; Minett et al., 2014) when sexual maturity is reached, but mice of this age are still undergoing extensive bone development. We used 12‐week‐old mice which have mature bone, defined by stable mechanical properties, bone mass and length (Ferguson et al., 2003). Moreover, at this stage the rate of bone mineralization and resorption reach a plateau. To our knowledge, this is the first study investigating the effects of CIBP in mice with fully developed bones.

In line with previous studies, we found a reduction in limb use and weight bearing on the affected limb in mice with bone cancer (Minett et al., 2014). There was a significant correlation between the two measures with the behavioural phenotypes deteriorating from intermediate stage cancer (defined by clear limping) to late stage cancer (where mice completely avoid use of the affected limb). Noteably, the extent of osteolytic lesions in both cortical and trabecular bone of the affected femur was more pronounced in intermediate stage cancer compared to late stage cancer mice. In accordance with a previous study (Falk et al., 2013), we found a positive correlation between bone degradation and skeletal pain. Our findings indicate a homeostatic equilibrium in bone degradation and/or new bone formation is already established when mice with bone cancer begin to reduce limb use (lower LSs that reflect limping), but does not progress further.

### 4.2 The role of osteoclasts in a model of RANKL‐induced bone resorption

RANKL can promote both osteoclastogenesis and recruitment of hematopoietic precursors from the bloodstream (Kotani et al., 2013). However, previous studies indicate that a single systemic injection of RANKL in mice is not sufficient to induce bone resorption (Tomimori et al., 2009). We therefore used the approach of intrafemoral injection of RANKL to induce activation of locally resident osteoclasts, both with and without a prior priming injection of systemic RANKL, but failed to observe evidence of dysregulated bone remodelling or pain behaviours. One possible explanation may be a dose‐limiting factor of the injected concentration of 10 μg RANKL, which may not be enough to induce sustained osteoclast activation. However, due to the instability of RANKL at higher concentrations and the maximum volume capacity of the mouse femur (Zilber et al., 2008), we were not able to administer a higher dose. Instead, we used a model of widespread bone resorption induced by systemic RANKL injections (Tomimori et al., 2009) to determine whether physiologic osteoclasts can induce pain behaviour in mice. Surprisingly, we found a significant decrease in BMD in male, but not female mice, 48 hr after two consecutive systemic RANKL injections. Previous work shows osteoclast activation in both male and female mice (Ariza et al., 2019; Ding et al., 2020; Li et al., 2015; Shin et al., 2014; Tomimori et al., 2009). One possibility for the discrepancy between our data and other studies may be related to a reduction in the accuracy of trabecular BMDs below 250 mg/cm^3^ when using two phantoms of calcium‐hydroxyapatite of 250 and 750 mg/cm^3^ to estimate density. 3D reconstructions and detailed analysis of bone microarchitecture, incuding bone volume fraction, bone surface fraction, trabecular number and connectivity density suggest some degree of bone resorption is present in female mice.

Even at higher systemic doses of RANKL, the significant decrease in BMD observed after 48 hr in male mice was not sustained throughout the experimental period of 20 days. These data reflects previous descriptions of recovery of normal bone turnover over a prolonged time period (Tomimori et al., 2009). However, using 3D reconstructions, we observed a trend of localized effects on bone resorption. When dividing the volumetric region in smaller segments of 0.2 mm thickness, we found significantly reduced BMD in the two segments located further away from the growth plate, which is likely due to differentiation of chondrocytes into osteoblasts near the growth plate, resulting in faster bone formation (Mizuhashi et al., 2019; Newton et al., 2019).

Bone strength is influenced by several factors, including material properties, geometry and notably tissue microarchitecture (Dalle Carbonare & Giannini, 2004; Felsenberg & Boonen, 2005). In humans, trabecular microarchitecture parameters correlate with fracture risk independently of BMD (Elizabeth et al., 2019; Kijowski et al., 2012). Similarly, in trabecular samples of the femoral head, bone microarchitecture parameters are better predictors of bone strength and structure than BMD (Topoliński et al., 2012). In this study, alongside reduction in BMD, we also observed significantly reduced bone surface fraction, trabecular number and connectivity density in mice following systemic RANKL administration that indicates osteoclast‐mediated bone resorption. Unexpectedly, trabecular thickness was significantly higher in RANKL‐treated mice, potentially reflecting a reduction in small trabeculae in these mice. Similar findings have been reported in an OVX rat model of bone resorption: in young rats oestrogen deprivation leads to reduced trabecular thickness but this effect is reversed with ageing (Butler et al., 2007). Similarly, in C57BL/6J mice ovariectomy produces a reduction or an increase in trabecular thickness for 8 week old, and 12 weeks or older animals, respectively (Zhou et al., 2018).

In mice with CIBP, we observed changes in both trabecular and cortical bone, reflecting tumour‐induced lesions (Bloom et al., 2011; Mantyh et al., 2010). Animals injected with RANKL, on the contrary, did not present any visible changes in cortical BMD. Therefore, the site of osteoclast activation may be important in determining pain development, as cortical lesions may sensitize periosteal afferents. However, sensitization of bone marrow afferents is also able to produce pain, as is observed in patients undergoing bone marrow aspiration (Gendron et al., 2019). Additionally, in osteoarthritic patients pain outcomes correlate with trabecular, but not cortical BMD (Burnett et al., 2017).

Overall, several trabecular bone microarchitecture parameters suggest significant alterations in bone microstructure in mice receiving two consecutive systemic injections of RANKL. However, the extent of osteoclast activation was not sufficient to induce pain behaviour, even at early timepoints when differences in BMD were more pronounced between RANKL‐ and vehicle‐injected mice. Compared to other inbred mouse strains, C57BL/6J have only few trabeculae and less mineralized bone (Zhou et al., 2018). Other mouse strains may therefore constitute a better model to study the effects of osteoclast activation and bone resorption.

### 4.3 Physiologic osteoclasts are not sufficient to induce skeletal pain

Although mice with late stage cancer and RANKL‐treated mice show similar reductions in BMD and bone microarchitecture parameters, pain behaviour was present only in mice with CIBP. The observed skeletal pain phenotype in late stage cancer mice may depend on a phenotypic switch in osteoclasts present in pathological conditions. Metastatic lung cancer to the bone produces ostolytic lesions by altering the bone microenvironment to promote the vicious cyle of bone resorption and tumour growth (Mundy, 2002). Some factors released in the bone microenvironemnt by cancer and stromal cells, including interleukin‐6 (IL‐6) and tumour necrosis factor α (TNF‐α) (Kim et al., 2009), are able to induce osteoclast differentiation in vitro independently of RANK activation (O’Brien et al., 2016). Blockade of IL‐6 in a rat model of bone cancer reduced mechanical hypersensitivity and ongoing pain, and prevented bone degradation, without affecting tumour burden (Remeniuk et al., 2018). Evidence from skeletal inflammatory conditions suggests that IL‐6 and TNF‐α induce ‘inflammatory osteoclasts’, with a distinct phenotypic profile that can be compared to different macrophage subtypes (Novack, 2016). In ovariectomized mice, femur marrows show increased levels of the cytokines TNF‐α, IL‐6, and interleukin‐1 β (IL‐1β) (Kanaya et al., 2016). Similarly, in a model of injury to the intervertebral disc, anti‐RANKL treatment reduces expression of TNF‐α and IL‐6 in the bone, while also preventing the increase of calcitonin gene‐related peptide (CGRP) in retrogradely labelled DRG neurones (Sato et al., 2015). In a serum‐transfer murine model of arthritis, pro‐inflammatory cytkoines including TNF‐α and IL‐6 are present in the joint (Ji et al., 2002). Additionally, both RANK knockout and wild‐type mice have osteoclasts in the arthritic synovium, although these cells are absent in the bone marrow compartment of knockout mice (O’Brien et al., 2016), suggesting a different origin from canonical osteoclastogeneis. It remains to be investigated if these so‐called ‘inflammatory osteoclasts’ consitute a distinct population from homeostatic osteoclasts in terms of gene expression and resorptive properties, but our findings support the notion that increased activity of physiologic ostecoclasts does not contribute to peripheral sensitization in bone pathologies.

## 5 CONCLUSION

In summary, this study reveals that physiologic osteoclasts alone are unable to induce skeletal pain in mice. Several painful skeletal conditions, including bone cancer, are associated with increased osteoclast activity, as well as neuronal sprouting, suggesting that several mechanisms may act in synchrony to produce and maintain chronic bone pain. One such mechanism may involve the phenotypic switch towards an ‘inflammatory osteoclasts’ mediated by TNF‐α and IL‐6.

## CONFLICT OF INTEREST

The authors report no conflict of interest.

## AUTHOR CONTRIBUTIONS

LdC, JW and SS conceived the study, designed the experiments and interpreted the results. LdC, SS and SSV performed the experiments. LdC analysed the data. LdC, SS and JW prepared the manuscript. All contributing authors discussed the results, commented on the manuscript and agreed to submission for publication.

## Supporting information

Fig S1

Methods S1
