# Supplementary material for: Physiologic osteoclasts are not sufficient to induce skeletal pain in mice
Source: Eur J Pain. 2020 Oct 12;25(1):199–212. doi: 10.1002/ejp.1662 (PMC8436750; doi:10.1002/ejp.1662)
Supplement: Supplementary file 1 — Fig S1 [file EJP-25-199-s002.pdf]

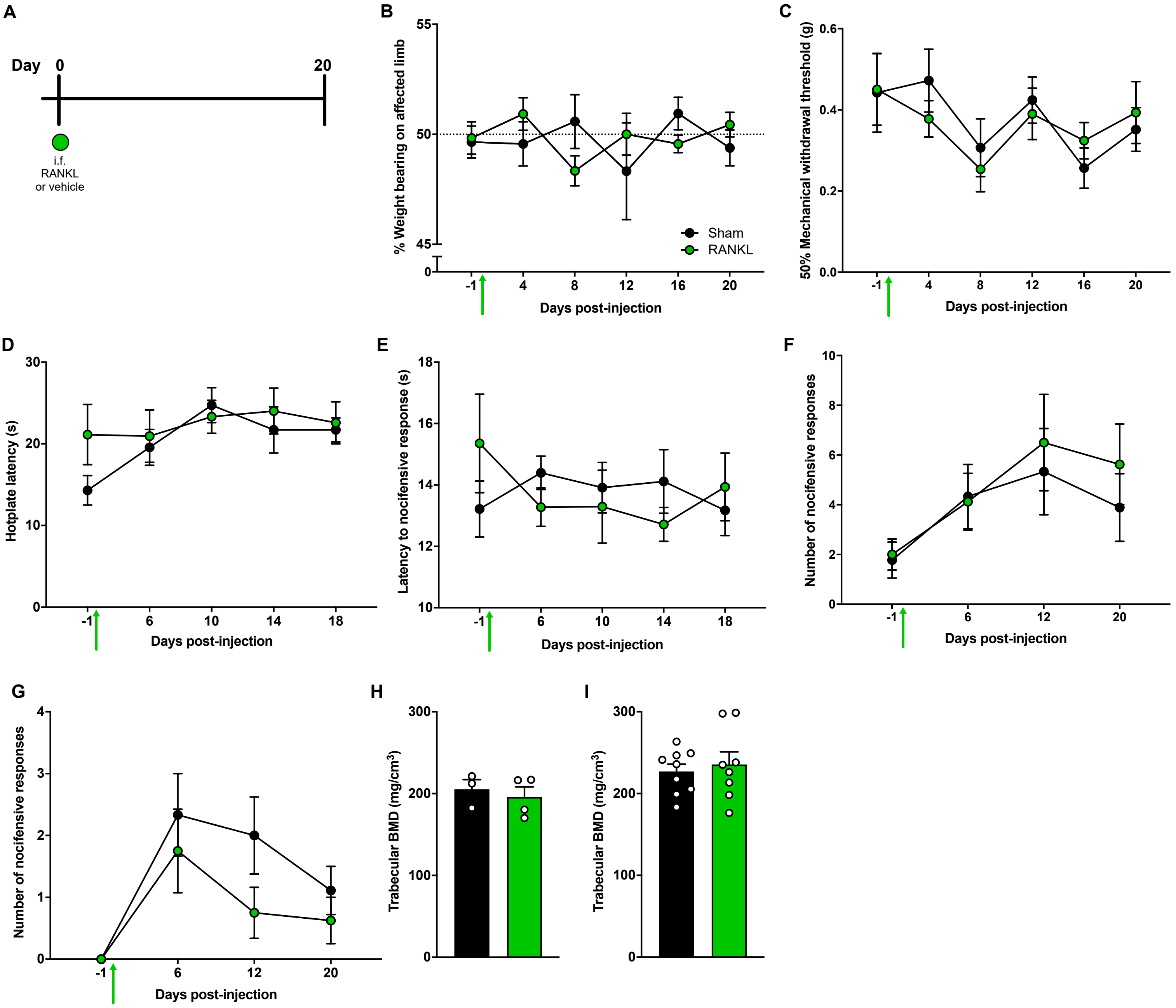

Figure S1. Effects of a single intrafemoral injection of RANKL on pain behavior and bone resorption. (A) Schematic overview of the study design. Mice received an intramedullary injection of RANKL (green, *n*=8) or vehicle (black, *n*=9) into the distal femur head (grey arrow) and behavior was closely followed for 20 days. (B) Mice were assessed for ongoing pain measured through weight bearing on the injected limb, (C) mechanical sensitivity to von Frey filaments, (D) heat sensitivity, and (E) cold sensitivity. (F) Nocifensive responses during a non-noxious 2 min palpation of the distal femur and (G) in a 2 min period afterwards were assessed. (H) Trabecular BMD in the ipsilateral femur at day 20. (I) Trabecular BMD 48 hr after post-injection (RANKL, *n*=4; sham, *n*=3). Data is shown as mean ± S.E.M.
