# Supplementary material for: Physiologic osteoclasts are not sufficient to induce skeletal pain in mice
Source: Eur J Pain. 2020 Oct 12;25(1):199–212. doi: 10.1002/ejp.1662 (PMC8436750; doi:10.1002/ejp.1662)
Supplement: Supplementary file 2 — Methods S1 [file EJP-25-199-s001.pdf]

Methods S1. Non-noxious palpation

Animals were gently restrained by one experimenter, while the other experimenter applied non-noxious pressure by holding the distal femur between thumb and index every 1 s for a total period of 2 min, as previously described (Sabino et al. 2003). The number of responses during the 2 min palpation and in the 2 min period afterwards, defined as attempted withdrawal and vocalizations were quantified.
